# Agile 3+ and Agile 4 scores are accurate to detect advanced metabolic dysfunction-associated steatotic liver disease

**DOI:** 10.1097/MEG.0000000000003113

**Published:** 2026-02-25

**Authors:** Muriel Manica, Luis F. Ferreira, Cristiane A. Villela-Nogueira, Edison R. Parise, Nathalie C. Leite, Ana C.F. Cardoso, Ana L.F. de Azevedo, Nilma L.S. Ruffeil, Cristiane V. Tovo

**Affiliations:** 1 School of Electronics, Electrical Engineering and Computer Science, Queen’s University of Belfast (QUB), Northern Ireland, United Kingdom

**Keywords:** advanced fibrosis, cirrhosis, elastography, liver steatosis, nonalcoholic fatty liver disease

## Abstract

**Background:**

The prognosis of metabolic dysfunction-associated steatotic liver disease (MASLD) is related to the presence of liver fibrosis (LF). The Agile 3+ and Agile 4 scores are proposed as noninvasive methods to identify advanced LF and cirrhosis, respectively, associating vibration-controlled transient elastography (VCTE) with clinical and laboratorial data.

**Aim:**

To evaluate the performance of Agile scores in the assessment of LF in MASLD patients.

**Methods:**

This is a cross-sectional multicentric study conducted at three gastroenterology/hepatology outpatient units from Brazil, including individuals with biopsy-proven MASLD. To calculate the Agile 3+ and Agile 4 scores for the evaluation of advanced fibrosis (F≥3) and cirrhosis (F4), respectively, the formula proposed by Sanyal *et al*. was applied.

**Results:**

A total of 220 patients were included, mostly women (*n* = 146; 66%) and with diabetes (*n* = 136; 61.5%). The prevalence of advanced fibrosis and cirrhosis was 21.82% (*n* = 48) and 9.55% (*n* = 21), respectively. There was a strong correlation between Agile 3+ (*r* = 0.751) and VCTE (*r* = 0.729) with the LF stage evaluated by liver biopsy, while the correlation of Agile 4 was moderate (*r* = 0.535). However, the Agile 3+ and Agile 4 scores have shown high sensitivity and specificity (higher than 80% in all cases) when compared to liver biopsy. Also, there was significance in the area under the ROC curve, which was inferior than 0.5 for all cases.

**Conclusion:**

A good correlation was confirmed between the Agile scores and VCTE with the stages of fibrosis in MASLD individuals, classifying correctly the presence of LF and cirrhosis better than VCTE solo, with a smaller indeterminate zone.

## Introduction

Metabolic dysfunction-associated steatotic liver disease (MASLD) [1] is the most prevalent cause of chronic liver disease in the West, with a world prevalence estimated at 25% [2]. The prognosis of MASLD is related to the presence of liver fibrosis (LF), with an important increase in mortality in those with advanced liver fibrosis (AF) [3,4].

Although liver biopsy (LB) is the current gold standard for the staging of LF, it must be postulated that it is an invasive procedure, with the risk of complications such as bleeding, and morbimortality that are not negligible; besides, the insufficient samples and variability of interpretation between the observers may occur [3,5]. In this context, many noninvasive methods have been developed and tested to predict LF, although with many limitations. The vibration-controlled transient elastography (VCTE), evaluating the liver stiffness measurements (LSM), is the most promising method and has been widely validated [6,7]. The Agile 3+ and Agile 4 scores have been recently proposed to identify advanced LF and cirrhosis, respectively, associating VCTE with clinical and laboratorial data [8,9].

This multicentric study aimed to compare the performance of Agile 3+ and Agile 4 scores with VCTE solo in the assessment of LF in patients with MASLD, compared with LB as a gold standard.

## Materials and methods

This is a cross-sectional multicentric study with prospective inclusion conducted at the outpatient units from three gastroenterology/hepatology Services in Brazil: the Clementino Fraga Filho University Hospital (Federal University of Rio de Janeiro – UFRJ), the São Paulo University Hospital (Federal University of São Paulo – UNIFESP), and the Santa Casa de Misericordia de Porto Alegre (Federal University of Health Sciences of Porto Alegre – UFCSPA), all reference tertiary hospitals.

All individuals who were at least 18 years old, with biopsy-proven MASLD, who attended the cited services in the last 10 years were included. Those with other concomitant liver disease etiologies and/or with insufficient data in the medical records were excluded.

MASLD was defined according to the new classification endorsed by the American Association for the Study of Liver Diseases, the European Association for the Study of the Liver, and the Associación Lationoamericana Para el Estudio del Higado [1].

The data collected from the medical records were demographic (sex, age, weight, height, and diabetes status), biochemical [platelets, total bilirubin, alanine aminotransferases (ALT) and aspartate aminotransferases (AST), total and low-density lipoprotein (LDL) cholesterol, and triglycerides], and data regarding LB and VCTE.

The BMI was calculated according to Nuttal *et al* [10], by dividing weight (in kilograms) by square height (in meters). The weight was assessed in a scale with a precision of 0.1 kg (Filizola, São Paulo, Brazil), and the stature in a stadiometer with a precision of 0.01 m (Cescorf, Porto Alegre, Brazil), with the patient standing, barefoot, and wearing the least clothes.

For the liver histopathology, percutaneous LB was performed guided by ultrasound, with 16-gauge needles. Experienced physicians obtained the fragments according to standard procedures [11]. A single experienced liver pathologist in each of the different centers, blinded to the study data, evaluated the LB specimens. The clinical research network in non-alcoholic steatohepatitis scoring system was applied to define steatosis, the presence of ballooning, lobular inflammation grades, non-alcoholic fat liver disease activity score, and fibrosis stage [12]. All included samples had at least 15 mm of length and at least 10 portal tracts.

Liver stiffness measurement was performed with FibroScan 502 touch (Echosens, Paris, France), using M or XL probes according to BMI (M probe was used in patients with BMI < 32 kg/m²), as proposed in the reference study [13]. A 4-hour fast was required to perform the examination. The FibroScan technique was previously described [14,15]. The final median is expressed in KiloPascal (kPa). Examinations with at least 10 valid measurements, interquartile range (IQR)/median liver stiffness ratio <30% and success rate >60%, were included in the analysis [16]. FibroScan was performed by a physician blinded to patients’ clinical and LB data.

Individuals included in the study were submitted to clinical and laboratory evaluation, LSM using VCTE and LB with a maximum interval between all the procedures of 2 weeks, according to FibroScan-AST study recommendations [13].

To calculate the Agile 3+, for the evaluation of advanced fibrosis (F≥3), the formula proposed by Sanyal *et al* [8] was applied, as follows:


$$Agile\;3+=\frac{e^{logit\left(\rho_{F\geq 3}\right)}}{1+e^{logit\left(\rho_{F\geq 3}\right)}}$$

Where: logit (pF > 3) = −3.92368 + 2.29714 × normal logarithm (transient elastography)  − 0.00902 × platelets − 0.98633 × ALT/AST Ratio-1 + 1.08636 × diabetes status − 0.38581 × sex + 0.03018 × age.

To calculate the Agile 4, for the evaluation of cirrhosis (F4), the formula proposed by Sanyal *et al* [8] was applied, as follows:


$$Agile\;4=\frac{e^{logit\left(\rho_{F=4}\right)}}{1+e^{logit\left(\rho_{F=4}\right)}}$$

Where: logit (pF = 4) = 7.50139 − 15.42498 × −0.01378 × platelets − 1.41149 × ALT/AST Ratio-1 − 0.53281 × sex + 0.41741 × diabetes status.

Both Agile 3+ and Agile 4 are predicted probabilities from the logistic regression model; the results vary between 0 and 1, and can be interpreted in a probabilistic way.

The project was approved at the Research Ethics Committee of Irmandade Santa Casa de Misericórdia de Porto Alegre (coordinator center), under approval letter number 982.654. Volunteers read and signed the Informed Consent Form. The entire research was conducted following Resolution 196/96 of the National Health Council (Brazil) and adhered to the principles of the Declaration of Helsinki for research involving human subjects. Data were processed in accordance with the General Data Protection Law (Brazilian Law Nº 13.709/2018).

In the statistical analysis, the description of qualitative variables was expressed in absolute and relative frequency. The results of the quantitative variables are presented in mean and SD, median, and IQR. Data were tested for normality by Shapiro-Wilk’s test, and the correlations were assessed by Spearman’s Correlation Coefficient (r_S_). The r_S_ was accepted as low when 0.1–0.29; moderate when 0.3–0.59; strong when 0.6–0.89, and very strong when >0.9.

The cutoff points were adopted following the most cited literature for LSM by VCTE [17], Agile 3+ [8], and Agile 4 [8,18] providing high sensitivity (Se) and specificity (Sp). Thus, the cutoffs used for advanced fibrosis were <0.351 and >0.679 for Agile 3+ [8] and <8 and >12 kPa [17] for LSM. For cirrhosis, the cutoffs were <0.169 and >0.388 for Agile 4 [18] and <10 and >15 kPa [17] for LSM.

In the analyses of advanced fibrosis and cirrhosis, applying the dual cutoffs, the proportion of patients with indeterminate scores, Se, Sp, positive predictive value (PPV), and negative predictive value (NPV) were calculated for VCTE, Agile 3+, and Agile 4 scores, in comparison to the LB results. The analyses have been done according to two methods, the first one being the inclusion of all individuals included, and the second with the exclusion of patients with indeterminate results (named ‘gray area’). The area under the ROC curve (AUROC) with the respective 95% confidence interval (95% CI) was calculated for all tests. The *P* value <0.05 was adopted as significant. The analyses were performed in the statistical software SPSS (IBM SPSS Statistics for Windows, Version 18.0, IBM Corp., Armonk, USA).

The statistical methods of this study were reviewed by the Statistical Committee of Universidade Federal de Ciências da Saúde de Porto Alegre (UFCSPA).

## Results

Two hundred and twenty-one patients were included, being mostly women (*n* = 146; 66%). There was a good homogeneity among groups when analyzing LF by LB (Table 1). The weight, height, BMI, and other blood test results were similar. However, the aminotransferases (ALT and AST) levels showed a statistically significant difference across the groups, being higher proportionally to the fibrosis stage.

**Table 1.** Basal characteristics of the total sample, and categorized by stage of fibrosis evaluated by liver biopsy

Continuous data mean±SD	Total sample			FIB 0			FIB 1			FIB 2			FIB 3			FIB 4			*P* value^a^
	(*n* = 220/100%)			(*n* = 61/27.6%)			(*n* = 90/40.72%)			(*n* = 21/9.5%)			(*n* = 27/12.22%)			(*n* = 21/9.5%)			
Age (years)	55.02	±	10.05	53.28	±	9.65	55.12	±	9.59	53.14	±	9.8	56.63	±	9.8	59.23		10.37	0.170
Weight (Kg)	84.46	±	16.03	85.93	±	16.61	86.22	±	16.62	77.29	±	13.42	88.39	±	13.42	74.44		10.92	0.083
Height (m)	1.6	±	0.09	1.6	±	0.1	1.59	±	0.1	1.62	±	0.06	1.59	±	0.06	1.6	±	0.1	0.875
BMI	32.18	±	5.29	32.73	±	5.26	32.61	±	5.5	29.93	±	5.33	32.67	±	5.33	30.42	±	3.7	0.131
TB	0.62	±	0.79	0.73	±	1.33	0.55	±	0.23	0.48	±	0.23	0.63	±	0.23	0.67	±	0.23	0.807
HDL	49.02	±	18.54	50.48	±	24.29	48.14	±	14.9	42.33	±	10.81	52.59	±	10.81	50.59	±	20.84	0.341
LDL	107.35	±	38.5	108.11	±	37.06	113.11	±	37.49	110.52	±	52.65	96.98	±	52.65	91	±	27.82	0.113
TC	189.44	±	41.43	195.47	±	37.43	192.1	±	39.77	184.43	±	62.23	179.19	±	62.23	179.59	±	39.73	0.353
TG	170.32	±	97.75	188.52	±	132.86	152.93	±	76.28	181.86	±	69.76	162.48	±	69.76	189.64	±	98.55	0.159
AST	35.55	±	22.86	29.66	±	17.8	32.31	±	17.66	39.05	±	22.7	44.19	±	22.7	51.23	±	41.12	0.000^b^
ALT	47.13	±	30.37	39.3	±	24.82	44.83	±	28.12	53.13	±	27.92	55.14	±	27.92	62.68	±	43.86	0.006^b^
VCTE	9.9	±	6.87	7	±	2.6	8.1	±	4.18	8.9	±	4.95	13.3	±	6.3	22.5	±	10.38	0.000^b^
Categoric Data - n (%)																			
SAH	94 (42.53)			34 (15.38)			36 (16.29)			7 (3.17)			8 (3.62)			9 (4.07)			0.712
DM	137 (61.99)			33 (14.93)			52 (23.53)			17 (7.24)			17 (6.33)			18 (8.14)			0.024^b^
Dyslipidemia	96 (43.44)			31 (14.03)			40 (18.1)			9 (4.07)			9 (4.07)			7 (3.17)			0.377
Met. syndrome	103 (46.61)			37 (16.74)			44 (19.91)			7 (3.17)			9 (4.07)			6 (2.71)			0.751

ALT, alanine aminotransferase; AST, aspartate aminotransferase; DM2, type 2 diabetes mellitus; FIB 0, no fibrosis; FIB 1, minimum fibrosis; FIB 2, moderate fibrosis; FIB 3, advanced fibrosis; FIB-4, cirrhosis; HDL, high-density lipoprotein cholesterol; LDL, low-density lipoprotein cholesterol; Met. Syndrome, Metabolic syndrome; n, sample; SAH, systemic arterial hypertension; TB, total bilirubin; TC, total cholesterol; VCTE, vibration-controlled transient elastography.

a Anova One Way for independent means.

b Statistically significant difference.

Also, almost 62% (*n* = 137) of the patients in this study had type 2 diabetes mellitus (DM2). In patients without any stage of LF, the prevalence of DM2 was 53% (*n* = 33), increasing along with higher fibrosis stages: F1 : 58% (*n* = 52), F4 : 86% (*n* = 18).

It is important to notice that cirrhotic patients had the lowest average values for weight, BMI, and LDL cholesterol and the highest for triglycerides, ALT, and AST (Table 1).

There was a strong correlation between the score of Agile 3+ and the fibrosis stage (*r* = 0.751) and between VCTE and fibrosis stage (*r* = 0.729) evaluated by LB. The correlation between the score Agile 4 and the stages of fibrosis was moderate (*r* = 0.535) (Table 2).

**Table 2.** Correlation analysis between stage of fibrosis (by liver biopsy) comparing Agile 3+ score, Agile 4 score, and vibration-controlled transient elastography

Stage of fibrosis	Sample	Agile 3+			Agile 4			VCTE		
	*n* (%)	Mean ± SD			Mean ± SD			Mean ± SD		
0	61 (27.73)	0.047	±	0.07	0.286	±	0.248	7.013	±	2.61
1	90 (40.91)	0.065	±	0.127	0.337	±	0.258	8.069	±	4.181
2	21 (9.55)	0.115	±	0.172	0.415	±	0.329	8.929	±	4.951
3	27 (12.27)	0.211	±	0.172	0.631	±	0.329	13.3	±	4.951
4	21 (9.55)	0.527	±	0.303	0.861	±	0.228	22.523	±	10.387
		*r*:.751^c^			*r*:.535^b^			*r*:.729^c^		
		*P*: < 0.001^a^			*P*: < 0.001^a^			*P*: < 0.001^a^		

Level 0, no fibrosis; 1, minimum fibrosis; 2, moderate fibrosis; 3, advanced fibrosis; 4, cirrhosis; n, sample; %: relative sample.

a Correlation statistically significant by Spearman’s *X*^2^ Correlation.

b Moderate Correlation.

c Strong Correlation.

In Table 2, it is also possible to notice that both Agile scores and the VCTE present higher values the more advanced the stage of fibrosis. In the Agile 3+ score, the mean value in patients with cirrhosis was 149% higher than in patients with advanced fibrosis. For VCTE, the cirrhotic patients have results three times higher when compared with the patients without advanced liver disease.

**Fig. 1.**
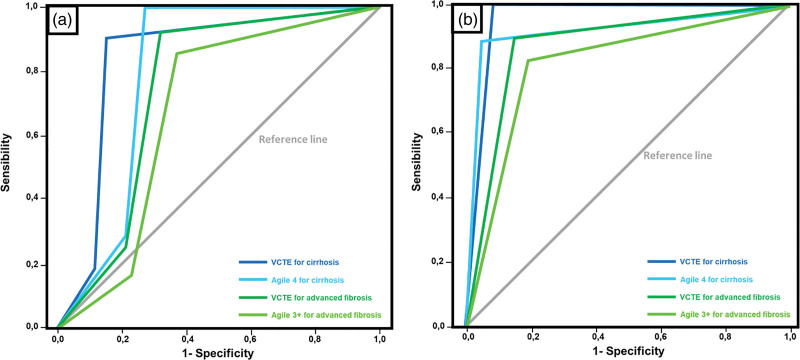
ROC Curves from VCTE, Agile 3+ and Agile 4 scores between patients with advanced fibrosis and cirrhosis diagnosed by liver biopsy. (a) Including the whole sample (*n* = 220); (b) Excluding patients on uncertain zones (excluded: *n* = 49/22.17% from VCTE for cirrhosis; *n* = 27/12.22% from Agile 4; *n* = 47/21.27% from VCTE for advanced fibrosis; *n* = 47/21.27% from Agile 3+). VCTE, vibration-controlled transient elastography.

Considering the analyses including all individuals (Table 3 and Fig. 1a), regarding AF, Agile 3+ presented better Se, Sp, PPV, and NPV than VCTE solo, although without a better AUROC. When analyzing cirrhosis, data are all favorable to Agile 4, instead of VCTE solo, including AUROC. Nevertheless, in this scenario of all individuals included in the analysis, both VCTE solo and Agile 4 NPV were small, which means an insufficient accuracy in excluding AF and cirrhosis in the sample of patients.

**Table 3.** AUROC for identification of fibrosis according to vibration-controlled transient elastography, Agile 3+, and Agile 4 scores between patients with advanced fibrosis or cirrhosis diagnosed by liver biopsy – Including all patients and excluding uncertain cases

	Diagnosis	n (%)	Method	Sensitivity	Specificity	AUROC	PPV	NPV	*P* value
						(CI 95%)			
Including all patients	Advanced fibrosis	220 (100%)	VCTE	86.03%	88.89%	0.741 (0.673–0.810)	96.69%	62.75%	<0.001
			Agile 3+	88.39%	90.63%	0.676 (0.601 –0.751)	97.06%	69.05%	<0.001
	Cirrhosis		VCTE	79.61%	71.43%	0.799 (0.741 –0.857)	95.28%	32.61%	<0.001
			Agile 4	86.84%	71.43%	0.838 (0.759 –0.916)	95.65%	42.86%	<0.001
Excluding uncertain patients	Advanced fibrosis	172 (77.83%)	VCTE	88.8%	87.7%	0.868 (0.799 –0.937)	66.6%	96.6%	<0.001
		174 (78.73%)	Agile 3+	82.5%	81.8%	0.822 (0.743 –0.900)	57.8%	93.9%	<0.001
	Cirrhosis	174 (78.73%)	VCTE	100%	91.0%	0.956 (0.927 –0.985)	51.7%	100%	<0.001
		194 (87.78%)	Agile 4	88.2%	94.8%	0.919 (0.811–1.000)	62.5%	98.8%	<0.001

%, relative sample included; AUROC, area under the ROC curve; CI, confidence interval; n, total sample included; *P*, statistical significance; PPV, positive predictive value; NPV, negative predictive value.

When the individuals with indeterminate results are excluded, the AUROC for Agile scores and VCTE solo are better for both AF and cirrhosis (Table 3 and Fig. 1b). In the evaluation of cirrhosis, Agile 4 and VCTE solo have shown AUROC > 0.9. However, the low PPV in this scenario limits the capacity to confirm AF and cirrhosis.

In the evaluation of AF, the indeterminate results were 21.27% for both Agile 3+ and VCTE solo. Regarding cirrhosis, the ‘gray area’ was 22.17% for VCTE solo and 12.22% for Agile 4.

As can be seen in Table 3, VCTE solo showed good statistical significance, high Se and Sp for diagnosing cirrhosis and advanced fibrosis in this sample of patients. Agile 4 has shown a better result in the detection of cirrhosis, including all patients, when compared to VCTE solo. In the remaining analyses, AUROC was better for VCTE solo.

## Discussion

The present study showed that Agile 3+ and Agile 4 have a good correlation with both VCTE and LB, presenting acceptable Se, Sp, and AUROC.

The scores Agile 3+ and Agile 4 were proposed to be used in the identification of AF and cirrhosis, respectively, and reduce the indeterminate results and the need of LB when compared to LSM by VCTE solo in patients with MASLD [8,9].

A recent study by Pennisi *et al*., [19] enrolling 1780 patients diagnosed with MASLD by LB has shown similar accuracy of Agile 3+ compared with LSM solo (AUROC = 0.88 for both of them). The same was found in Oeda *et al*. [20] study, with 641 patients, when no differences were found between the Agile scores and VCTE, with good AUROC for both scores. However, critical differences between the analyses in both studies, and the one presented here, can be found. Pennisi *et al* [19], in their study, calculated new cutoff points based on their sample, decreasing the number of individuals on the indeterminate zone, which may lead the result to a higher significance; whereas Oeda *et al* [20] just excluded the patients in the indeterminate zone from the analyzes (more than a third of the sample), artificially reaching good results.

For all scores, like Agile scores, FIB-4, and others, that aim to diagnose liver disease, avoiding the LB, a big problem is the area that is neither below the rule-out cutoff, nor above the rule-in cutoff (named ‘grey area’). For example, Sanyal *et al*. [8] in the original validating study, suggested, for Agile 3+ scores <0.451 to rule-out, and ≥0.679 to rule-in, and for Agile 4 < 0.251 to rule-out and ≥0.565 to rule-in. This large area ends up resulting in uncertainty about the diagnosis. These different analyses (whether or not including patients in the gray area) can end up causing studies to reach different outcomes, directly impacting clinical practice.

Papatheodoridi *et al* [18], in their study, after directly requesting the original authors [8], adjusted cutoffs for Agile 4, providing 90% Se and Sp (<0.169 and >0.388). The comparison, then, was between tests with the same theoretical Se and Sp.

In the present study, analyzing all patients included, Agile 4 has shown a smaller indeterminate area when compared with VCTE solo in the identification of cirrhosis, similar to Papatheodoridi *et al* [18] study. The same result did not occur in AF patients, in which the grey area was similar between Agile 3+ and VCTE solo.

Also, in the present study, analyzing all individuals included in the study, adopting Papatheodoridi’s cutoffs for AF, Agile 3+ had an indeterminate zone of 21.1% and the AUROC was 0.676. These results were not better than LSM by VCTE (Table 3), but were almost similar to what was found in Papatheodoridi’s [18] study. For cirrhosis, Agile 4 presented a smaller indeterminate zone (12.2%), similar results compared with Papatheodoridi’s [18], and a higher AUROC (0.838) than LSM solo.

It must be noted that, following the same kind of analysis made by Oeda’s [20] study, excluding the indeterminate zone in the analysis of sensibility and Sp, there is a better accuracy in the results for both VCTE and Agile scores when using the original cutoffs [21] and also the adjusted ones (Table 3). Nevertheless, in clinical practice, this proportion of patients in the ‘grey area’ must be considered in the interpretation of the results from noninvasive methods for LF. This is the reason why, even with less promising results, the analyses that include all individuals are the most trustworthy in the evaluation of some method accuracy.

The present study was a multicenter study on a multiethnic Brazilian population, which can be very different from studies in populations more homogeneous, like the Japanese population [20], or even in studies in a single center. It means that the differences in the results found in this study may be explained by the differences in the data used to calculate the Agile scores.

It is possible to notice that the sample of the present study had a mean age lower than Oeda’s [20] and Miura’s [22] studies; however, it was almost the same as Sanyal’s, Pennisi’s, and Papatheodoridi’s study [8,18,19,23]. The present study had lower levels of aminotransferases than Sanyal’s [8] and Oeda’s studies [20]. The prevalence of DM2 in our study (61.5%) was higher than Oeda’s [20] (54.9%), Sanyal’s [8](45.8–51%), and Papatheodoridis’s [18] (41.8%). However, Pennisi *et al* [19] conducted their work only with diabetic patients.

Although those data are directly connected to the Agile scores results, the formulas have this exact function: to correct possible inequalities in the sample’s distributions. But, as those formulas are still new, and were not tested in multiple populations, we still need to clarify if some corrections are needed for populations, for example, with low DM2 prevalence, such as Sanyal *et al* [8], or higher proportions, like Pennisi’s study [23].

Regarding the stages of LF, in the original study, Sanyal *et al* [8] found a prevalence of 37% for AF and 13% for cirrhosis. Those percentages may affect the PPV and NPV calculation. In this scenario, the PPV found was 81% and 63% for Agile 3+ and Agile 4, respectively, and the NPV found was 90% and 97% for Agile 3+ and Agile 4, respectively. Comparing to the original study, Papatheodoridi *et al* [18] found a lower prevalence for both AF (28.5%) and cirrhosis (11.8%), and the PPV found was 65% and 48% for Agile 3+ and Agile 4, respectively, and the NPV found was 95% and 97% for Agile 3+ and Agile 4, respectively. As expected, the lower prevalence of AF and cirrhosis reduced the PPV and increased the NPV.

In the present study, the prevalences of AF and cirrhosis, proven by LB, were 21.72% and 9.55%, respectively, smaller than the other comparable papers [8,18,20,22,23]. When analyzing AF, the PPV was 97.06% and 96.69% and the NPV was 69.05% and 62.75% for Agile 3+ and VCTE solo, respectively. In cirrhosis investigation, the PPV was 95.65% and 95.28% and the NPV was 42.86% and 32.61% for Agile 4 and VCTE solo, respectively. It means that both Agile scores and VCTE solo are more useful in confirming AF and cirrhosis and less reliable in excluding them.

The VCTE is the most validated noninvasive method to exclude advanced LF, demonstrating high sensibility and Se [24]. However, limitations exist, with reduced reproducibility in patients with large steatosis, elevated BMI, and early fibrosis. A systematic review with meta-analysis [25] evaluated 82 studies (14 609 patients), showing that the accuracy for diagnosing advanced fibrosis in patients with MASLD is 85%. Another meta-analysis [26] included data from 37 studies (5735 patients) evaluating the diagnostic performance of VCTE in MASLD, showing an AUROC of 0.85 in the diagnosis of advanced fibrosis. Supporting this information, a prospective multicentric study[4] evaluated 450 adults, in which LSM by VCTE identified patients with fibrosis with an AUROC of 0.80 for fibrosis ≥F3, and 0.89 for fibrosis ≥F4. In the most recent study from the LITMUS Project, VCTE showed acceptable accuracy with an AUROC of 0.83[16]. Notably, the results with VCTE were less significant in detecting MASLD and fibrosis (AUROC 0.61), similar to the FIB-4 score. For the present study, AUROC was 0.741 for AF and 0.799 for cirrhosis.

This study has some limitations. Although it is a multicenter study, the number of participants is relatively small when compared to previous studies. In consequence, with a smaller proportion of patients with AF and cirrhosis, it is more difficult to achieve statistically significant results. Also, once this is a retrospective real-life study, the LB were analyzed by a single pathologist in each center, and the records were collected in the medical registry.

Another limitation, not specific to this study, but to this area of knowledge, is the lack of validation studies for Agile 3+ and Agile 4, because they are new scores, published for the first time in 2021[9], and widely discussed in the literature since 2023[8]. So, studies like ours are very important to advance the knowledge about those scores, testing them in different populations.

In conclusion, the present study confirmed a good correlation between the Agile scores and VCTE with the stages of fibrosis in individuals with MASLD. Also, there was good reliability in AF and cirrhosis. Agile 4 classified correctly the presence or absence of cirrhosis better than VCTE solo in this population, with a smaller indeterminate zone.

## Acknowledgements

This study was financed in part by the Coordenação de Aperfeiçoamento de Pessoal de Nível Superior – Brasil (CAPES) – Finance Code 001.

### Conflicts of interest

There are no conflicts of interest.
